# Multidimensional financial hardship among uninsured and insured young adult patients with metastatic breast cancer

**DOI:** 10.1002/cam4.5885

**Published:** 2023-05-06

**Authors:** Stephanie B. Wheeler, Jennifer C. Spencer, Michelle L. Manning, Cleo A. Samuel, Katherine E. Reeder‐Hayes, Rachel A. Greenup, Lisa P. Spees, Donald L. Rosenstein

**Affiliations:** ^1^ Department of Health Policy and Management, Gillings School of Global Public Health University of North Carolina at Chapel Hill Chapel Hill North Carolina USA; ^2^ Lineberger Comprehensive Cancer Center University of North Carolina at Chapel Hill Chapel Hill North Carolina USA; ^3^ Duke University School of Medicine Durham North Carolina USA; ^4^ Department of Psychiatry, School of Medicine University of North Carolina at Chapel Hill Chapel Hill North Carolina USA; ^5^ Present address: Department of Surgery (Oncology) Yale University New Haven Connecticut USA

## Abstract

**Background:**

Little is known about the heterogeneous nature of financial hardship in younger patients with metastatic disease and the extent to which insurance protects against it. We examine the association between insurance status and multidimensional indicators of financial hardship in a national sample of women with metastatic breast cancer.

**Methods:**

We conducted a national, retrospective online survey in partnership with the Metastatic Breast Cancer Network. Eligible participants were ≥18 years, diagnosed with metastatic breast cancer, and able to respond in English. We estimated multivariate generalized linear models predicting two distinct dimensions of financial hardship—financial insecurity (the ability to afford care and living costs) and financial distress (the extent of emotional/psychological distress experienced due to costs)—as a function of insurance status.

**Results:**

Participants responded from 41 states (*N* = 1054; median age: 44 years). Overall, 30% were uninsured. *Financial insecurity* was more frequently reported by uninsured respondents. In adjusted analyses, uninsured participants were more likely than insured participants to report contact by debt collectors (adjusted risk ratio [aRR]: 2.38 [2.06, 2.76]) and being unable to meet monthly expenses (aRR: 2.11 [1.68, 2.66]). *Financial distress* was reported more frequently by insured participants. For example, insured participants were more likely to worry about future financial problems due to cancer and distress about lack of cost transparency. After adjustment, uninsured participants remained about half as likely as insured participants to report financial distress.

**Conclusions:**

Young adult women with metastatic cancer reported a high burden of financial toxicity. Importantly, insurance does not protect against financial distress; however, the uninsured are the most materially vulnerable.

## 1 BACKGROUND

The increasing cost burden among patients with cancer threatens to destabilize substantial scientific advancements in cancer prevention and control by rendering effective medications and other treatments unaffordable and inaccessible. The adverse financial impact of cancer, also known as “financial toxicity,”^1^ is a product of both high material burden and significant financial distress resulting from cancer care costs.^2^ Both financial insecurity and financial distress can lead to behavioral responses that negatively influence patients' and caregivers' health outcomes,^3^ such as delaying or foregoing medically necessary treatments and changing household consumption patterns (e.g., choosing to pay for medications instead of food).^4^ Moreover, the proliferation of cost‐related barriers to cancer care may limit access to treatment and exacerbate existing disparities in cancer outcomes.^5^ Among young women with breast cancer especially, the proliferation of high‐deductible health plans and shifting costs to patients has led to substantially higher cost burdens and treatment delays.^6^, ^7^

Relatively little is known about the financial impact of cancer treatment in patients with advanced or incurable disease, despite the high cost of their care.^8^ Moreover, women with advanced breast cancer generally have lower health insurance literacy than their early‐stage counterparts, which may affect their ability to optimize health insurance benefits when available.^9^ Low‐income, uninsured or publicly insured, and Black women are more likely than high‐income, privately insured, and White women, respectively, to be diagnosed with advanced breast cancer.^10^ They are also more vulnerable to financial hardship following treatment for advanced cancer.^11^ It is important to understand the extent and nature of financial insecurity and financial distress among patients with metastatic disease to better design and provide interventions for those patients most in need and to reduce access barriers that may contribute to outcome disparities.^12^

Prior studies have documented higher out‐of‐pocket material burden among uninsured (vs. insured) people with cancer.^13^, ^14^ But, to our knowledge, no studies have explored whether the extent and nature of financial insecurity and financial distress differ by insurance status among younger patients with advanced disease. Because health insurance expansion and insurance plan design are important mechanisms through which the financial consequences of a cancer diagnosis can be mitigated,^6^ we examined the association between insurance status and adverse financial outcomes in a national sample of young women with metastatic breast cancer.

## 2 METHODS

### 2.1 Participants

We partnered with the Metastatic Breast Cancer Network (MBCN) to field an online survey of network members over a 14‐day period in 2018 using Qualtrics. The survey invitation was emailed and posted through the MBCN and required approximately 20 min to complete. Participants were offered a $10 digital gift card as compensation; digital delivery of the gift card via email was logistically straightforward to administer and reduced time to compensation for participants. Survey items included sociodemographic information, health insurance status, cost‐related communication with providers, posttreatment financial burden, emotional well‐being, and financial coping strategies. A total of 1691 responses were received. Responses were removed if fewer than 50% of all survey items were complete or if responses were determined to be duplicates—either through the use of identical email addresses or through providing identical responses to a series of 13 demographic variables, including both multiple‐choice and free‐text responses. A total of 1054 (62%) unique responses were analyzed. All study activities were approved the Institutional Review Board at the University of North Carolina at Chapel Hill.

### 2.2 Measures

#### 2.2.1 Dependent variables

Financial toxicity is a multifaceted concept, with several existing definitions.^15^ Following a model proposed by Altice et al.^16^ we considered two distinct aspects of financial hardship: *financial security*—the ability to afford the material costs associated with cancer care and living expenses and *financial distress*—the extent to which individuals experience psychological distress as a direct result of cancer care costs. Eleven items were taken from the COmprehensive Score for financial Toxicity (COST).^17^ As the COST composite score combines multiple domains of financial toxicity into an aggregate score, it is more informative for this analysis to examine the individual items rather than the composite score, although we present both. We also created survey‐specific measures to further describe each domain. To further assess financial insecurity, we asked whether participants had been contacted by collections or had filed for bankruptcy as a result of their cancer costs. To further assess financial distress, we evaluated two statements with 5‐point Likert scale responses: “I have been distressed by not knowing what my cancer care costs would be” and “I am worried about the financial stress on my family as a result of my cancer”. All questions are listed with response options in Table S1.

### 2.3 Analysis

For Likert‐scaled financial distress statements, responses were dichotomized. Responses were categorized as high distress when subjects indicated that they were worried by or anxious about costs “quite a bit” or “very much”. Financial insecurity was indicated by answering “not at all” or “a little bit” to Likert‐scaled statements regarding financial security such as, “I am able to meet my monthly expenses.” Questions assessing treatment refusal and contact from collections were answered as yes/no, with affirmative responses indicating financial insecurity.

#### 2.3.1 Independent variables

Participants were asked whether they currently had any health insurance and those who responded “no” were categorized as uninsured. For those who responded yes, a mutually exclusive insurance type was coded as individuals reporting: any Medicaid; any Medicare without Medicaid dual enrollment; private insurance; or other health insurance (e.g., TriCare). Insured individuals were also asked whether their insurance covered prescription drug medications “well,” “some,” or “not at all.” Other sociodemographic variables included current age, race/ethnicity (coded as non‐Hispanic/Latina White, Black, Hispanic/Latina, and other), years living with metastatic disease, whether one or more dependent lives in the household, total household income, educational attainment, employment, and marital status.

### 2.4 Analyses

We report financial outcomes stratified by health insurance status. Differences between insured and uninsured individuals were assessed using a Wald test. We estimated multivariable regression models predicting financial insecurity and financial distress as a function of insurance status, adjusting for age, race/ethnicity, time since metastatic diagnosis, presence of dependents in the household, household income, education, and marital status. Regressions were specified as a generalized linear model (GLM) with a Poisson distribution, log link, and robust standard errors, resulting in an adjusted relative risk of each outcome. All analysis was performed with Stata 15.

## 3 RESULTS

### 3.1 Participant characteristics

Participants (*N* = 1054) represented 41 states. Uninsured participants (*n* = 316; 30.0%) were more likely than insured participants (*n* = 738; 70.0%) to be under age 40 (69.6% vs. 55.3%, *p* < 0.001). Uninsured participants were much less likely to be non‐Hispanic/Latina White (38.3% vs. 78.0%, *p* < 0.001). Among the uninsured, 8.5% reported Hispanic/Latina ethnicity, 22.8% reported Black race, and 30.4% reported another race or ethnicity. Compared with insured participants, uninsured participants reported living with metastatic disease for a longer period, with 40.5% of uninsured participants living with metastatic disease for 2 years or more compared with 24.2% of insured participants (*p* < 0.001). Uninsured participants reported lower average household income and educational attainment, but were much more likely to be working, with 73.7% working full time (compared with 27.2% of insured participants) and 18.0% working part time (compared with 8.3% of insured participants). Among those who were insured, the majority reported private insurance (54.7%) followed by Medicare excluding dual enrollment with Medicaid (24.1%), Medicaid (including dual enrollment) (18.0%), and other insurance sources (3.1%) (Table 1).

**TABLE 1:** Demographic characteristics by insurance status.

	Insured	Uninsured	*p* value
	*n* = 738 (70%)	*n* = 316 (30%)	
Age (mean)	44.0 (34.0–48.0)	43.0 (39.0–47.0)	—
Race			
White	576 (78.0%)	121 (38.3%)	<0.001
Black	23 (3.1%)	72 (22.8%)	
Hispanic/Latina	43 (5.8%)	27 (8.5%)	
Other	96 (13.0%)	96 (30.4%)	
Years with metastatic disease			
<1 year	211 (28.6%)	37 (11.7%)	<0.001
1–2 years	348 (47.2%)	151 (47.8%)	
2–5 years	153 (20.8%)	106 (33.5%)	
5+ years	25 (3.4%)	22 (7.0%)	
Marital status			
Divorced/widowed/separated	32 (4.4%)	46 (14.6%)	<0.001
Married, or living with a partner	544 (74.0%)	252 (79.7%)	
Never married	159 (21.6%)	18 (5.7%)	
Dependents in the household			
No	83 (11.3%)	17 (5.4%)	0.003
Yes	654 (88.7%)	299 (94.6%)	
Household income			
<15,000	13 (1.8%)	38 (12.0%)	<0.001
15,000–29,999	87 (11.9%)	87 (27.5%)	
30,000–49,999	399 (54.7%)	153 (48.4%)	
50,000 or more	231 (31.6%)	38 (12.0%)	
Educational attainment			
High school or less	245 (33.2%)	32 (10.1%)	<0.001
Some college, no degree awarded	190 (25.8%)	142 (44.9%)	
Two‐year degree/vocational school	103 (14.0%)	116 (36.7%)	
Four‐year degree or higher	199 (27.0%)	26 (8.2%)	
Working currently			
No	448 (60.9%)	10 (3.2%)	<0.001
Yes	288 (39.1%)	306 (96.8%)	
Work structure (*n* = 594)			
Full time	200 (69.4%)	233 (76.1%)	0.085
Part time	61 (21.2%)	57 (18.6%)	
Self employed	27 (9.4%)	16 (5.2%)	
COST total	26.3 (6.5)	22.3 (6.6)	<0.001
Insurance type (*n* = 738)			
Private	404 (54.7%)		<0.001
Medicaid	133 (18.0%)		
Medicare	178 (24.1%)		
Other	23 (3.1%)		
Prescription coverage (*n* = 738)			
No coverage	29 (4.0%)		
“Covers them some”	466 (64.9%)		
“Covers them well”	223 (31.1%)		

*Note*: COST, comprehensive score for financial toxicity, range (0–44); higher scores indicate worse toxicity; differences assessed using *X* ^2^ test for categorical variables and *t*‐tests for continuous variables.

### 3.2 Financial insecurity

**FIGURE 1:**
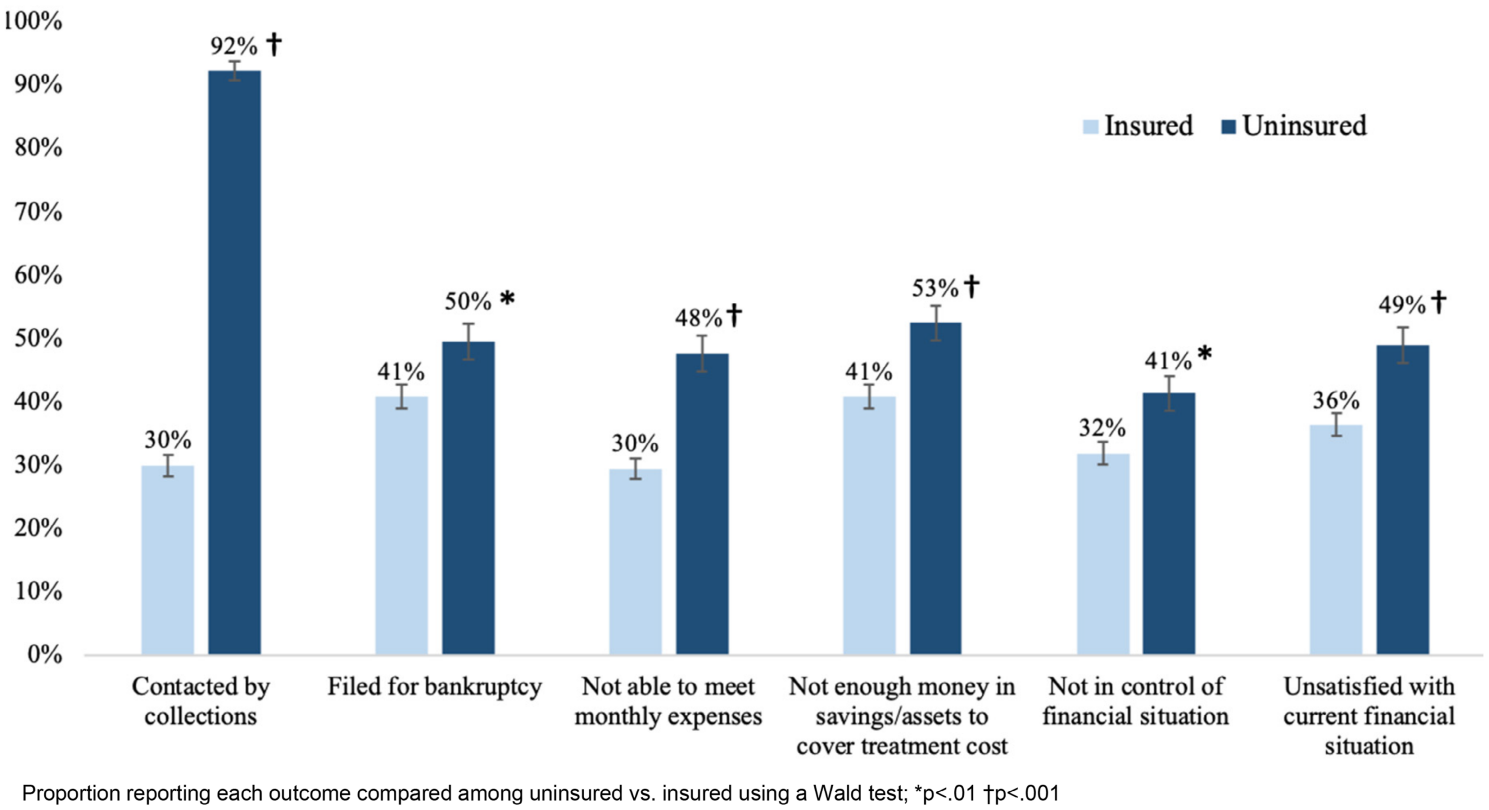
Unadjusted prevalence of financial insecurity by insurance status.

We observed a high prevalence of financial insecurity reported across all participants, but prevalence was higher among the uninsured relative to the insured (Figure 1). Notably, 92% of uninsured participants reported having been contacted by debt collectors compared with 30% of insured participants (*p* < 0.001), and 50% of uninsured versus 41% of insured participants reported filing for bankruptcy as a result of their cancer (*p* = 0.008). Half of uninsured participants reported that they were unable to meet their monthly expenses (48%) or did not have enough money in savings or assets to cover the cost of their treatment (53%). While this proportion was lower among insured participants, it still represented a large share of responses, with 30% of insured participants reporting that they were unable to meet monthly expenses, and 41% reporting that they did not have enough money to cover the cost of treatments. Participants also reported general concerns with their financial status as a result of cancer, with 41% of uninsured and 32% of insured participants reporting they did not feel in control of their financial situation (*p* = 0.003), and 49% of uninsured and 36% of insured participants reporting that they were dissatisfied with their current financial situation (*p* < 0.001).

In adjusted analyses (Table 2), uninsured participants remained more likely than insured participants to report being contacted by debt collectors (adjusted risk ratio aRR:2.38 [2.06, 2.76]), being unable to meet monthly expenses (aRR:2.11 [1.68, 2.66]), and feeling out of control of (aRR:1.71 [1.36, 2.14]), or dissatisfied with (aRR:1.82 [1.50, 2.21]), their current financial situation. The strongest consistent predictor of financial insecurity was household income, with people reporting household incomes under $50,000 per year at increased risk of financial insecurity. Time since diagnosis also was predictive of outcomes; specifically, participants who were 1–2 years postmetastatic diagnosis reported greater financial insecurity than participants who were in the first year.

**TABLE 2:** Adjusted relative risk of financial insecurity.

	Contacted by collections		Filed for bankruptcy		Unable to meet monthly expenses		Not enough in savings or assets to cover cost of cancer		Not in control of financial situation		Unsatisfied with current financial situation	
	RR	95% CI	RR	95% CI	RR	95% CI	RR	95% CI	RR	95% CI	RR	95% CI
Uninsured (ref: insured)	**2.38**	**[2.06, 2.76]**	1.09	[0.92, 1.30]	**2.11**	**[1.68, 2.66]**	1.07	[0.91,1.27]	**1.71**	**[1.36, 2.14]**	**1.82**	**[1.50, 2.21]**
Age (years, ref: 40+)												
Under 40	**0.74**	**[0.64, 0.86]**	**1.79**	**[1.54, 2.09]**	**0.53**	**[0.41, 0.67]**	**1.34**	**[1.11, 1.61]**	**0.38**	**[0.29, 0.49]**	**0.41**	**[0.33, 0.51]**
Race (ref: White)												
Black	**0.80**	**[0.66, 0.96]**	**1.49**	**[1.14, 1.94]**	1.07	[0.78, 1.48]	0.96	[0.72, 1.26]	1.14	[0.83, 1.55]	0.86	[0.64, 1.15]
Hispanic/Latina	**0.60**	**[0.47, 0.75]**	**1.99**	**[1.51, 2.61]**	**0.58**	**[0.37, 0.91]**	**1.61**	**[1.26, 2.07]**	0.75	[0.50, 1.12]	**0.71**	**[0.53, 0.96]**
Other	**0.70**	**[0.59, 0.82]**	**0.69**	**[0.50, 0.96]**	**0.49**	**[0.34, 0.71]**	0.85	[0.67, 1.07]	**0.37**	**[0.25, 0.56]**	**0.32**	**[0.23, 0.46]**
Years w/ metastatic disease (ref:<1)												
1–2 years	**1.61**	**[1.36, 1.90]**	**0.47**	**[0.41, 0.55]**	**1.38**	**[1.14, 1.67]**	**0.67**	**[0.57, 0.79]**	**1.33**	**[1.10, 1.62]**	**1.31**	**[1.09, 1.58]**
2–5 years	1.13	[0.93, 1.36]	**0.66**	**[0.54, 0.80]**	1.05	[0.80, 1.39]	**0.60**	**[0.48, 0.74]**	1	[0.76, 1.31]	1.06	[0.82, 1.35]
5+ years	**1.44**	**[1.13, 1.82]**	**0.41**	**[0.24, 0.71]**	1.09	[0.67, 1.77]	1.02	[0.77, 1.36]	0.98	[0.61, 1.60]	1.17	[0.80, 1.73]
Dependent in household	**1.59**	**[1.05, 2.41]**	**3.81**	**[1.92, 7.55]**	0.82	[0.61, 1.10]	1.18	[0.83, 1.68]	**0.65**	**[0.51, 0.84]**	**0.69**	**[0.56, 0.85]**
Annual household income (ref:50,000+)												
<15,000	**2.29**	**[1.72, 3.05]**	**4.69**	**[2.48, 8.87]**	**1.95**	**[1.21, 3.14]**	**1.74**	**[1.18, 2.56]**	**1.95**	**[1.33, 2.86]**	**1.64**	**[1.17, 2.28]**
15,000‐29,999	**2.59**	**[2.01, 3.33]**	**5.90**	**[3.46, 10.08]**	**1.50**	**[1.04, 2.16]**	**2.09**	**[1.63, 2.68]**	1.32	[0.93, 1.87]	1.09	[0.82, 1.45]
30,000‐49,999	**2.12**	**[1.66, 2.70]**	**5.91**	**[3.54, 9.89]**	**1.79**	**[1.31, 2.45]**	**1.66**	**[1.30, 2.12]**	**1.44**	**[1.10, 1.89]**	1.19	[0.96, 1.48]
Education (ref: 4‐year college degree)												
High school or less	1.17	[0.89, 1.52]	1.14	[0.86, 1.50]	**2.19**	**[1.55, 3.08]**	**0.43**	**[0.31, 0.58]**	**1.41**	**[1.05, 1.90]**	**1.44**	**[1.12, 1.84]**
Some college, no degree awarded	**1.46**	**[1.16, 1.82]**	1.17	[0.89, 1.55]	1.05	[0.73, 1.51]	**0.79**	**[0.64, 0.99]**	0.71	[0.51, 1.00]	0.85	[0.65, 1.12]
Two‐year degree/vocational school	**1.64**	**[1.31, 2.05]**	**1.42**	**[1.09, 1.84]**	1.07	[0.73, 1.58]	1.09	[0.87, 1.35]	0.83	[0.59, 1.18]	0.93	[0.69, 1.25]
Marital status (ref: unmarried)												
Married, or living with a partner	**1.32**	**[1.15, 1.52]**	**1.44**	**[1.12, 1.85]**	0.92	[0.72, 1.17]	**1.63**	**[1.30, 2.03]**	1.15	[0.90, 1.47]	0.96	[0.78, 1.17]

*Note*: Adjusted risk ratio from generalized linear model (*N* = 1054). Bolded values represent the relative risk of reporting a domain is significantly different from the referent group at *p* < 0.05.

### 3.3 Financial distress

**FIGURE 2:**
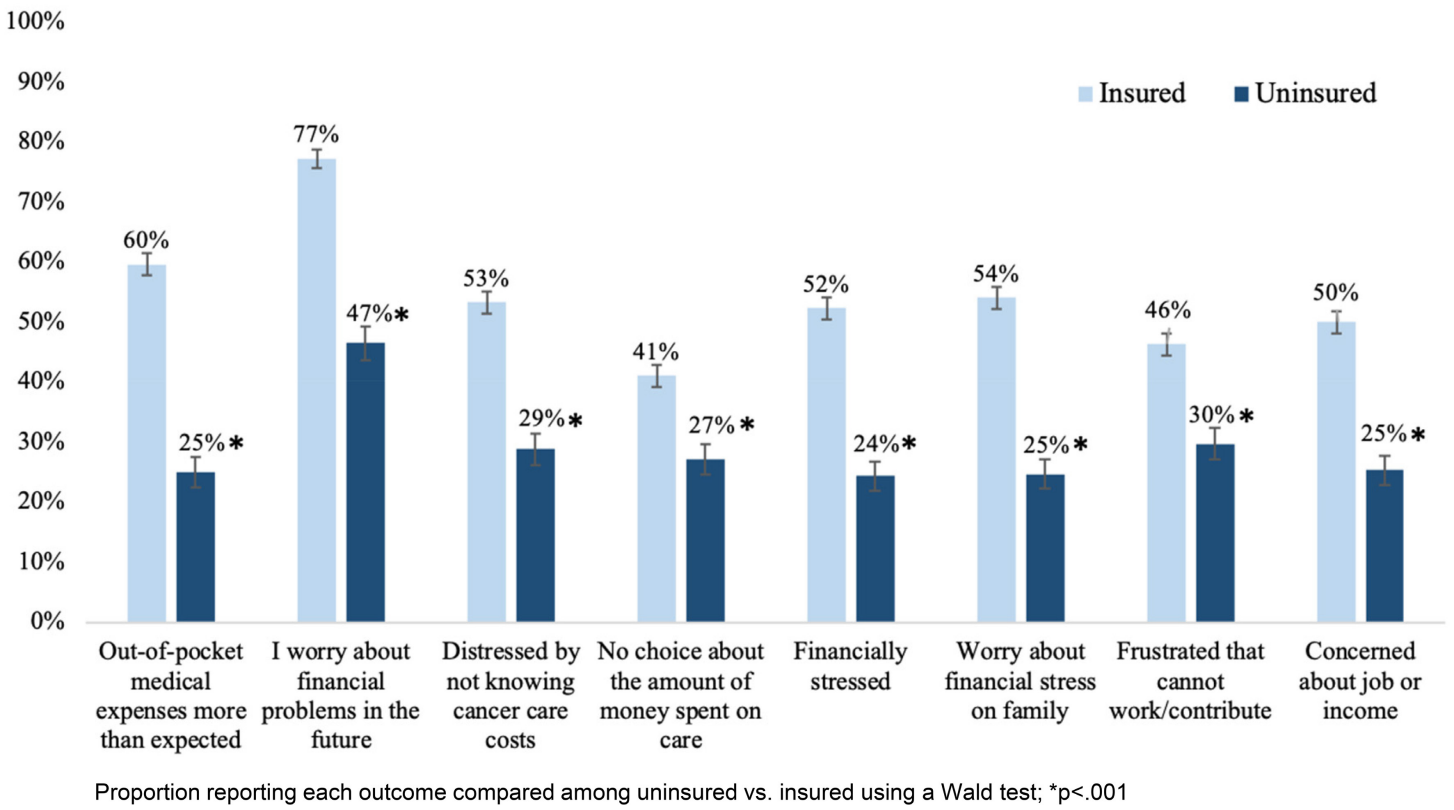
Unadjusted prevalence of financial distress by insurance status.

Financial distress was also prevalent among all participants; however, in sharp contrast to the higher rates of financial insecurity reported among the uninsured, insured participants consistently reported higher financial distress than their uninsured counterparts (Figure 2). One‐fourth of all uninsured participants reported that their out‐of‐pocket medical expenses were higher than expected (25%) compared with 60% of insured participants (*p* < 0.001). Uninsured participants were also less likely than insured participants to report worrying about financial problems in the future as a result of cancer (47% vs. 77%, *p* < 0.001) or about the impact of financial stress on their family (25% vs. 54%, *p* < 0.001). For uninsured participants, 29% reported being distressed by not knowing what their cancer care costs would be, compared to more than half of insured participants (53%, *p* < 0.001). When asked about the amount of money they spend on care, 27% of uninsured participants felt they had no control of the amount spent compared to 41% of insured women (*p* < 0.001). Finally, uninsured participants were less likely to be concerned about employment changes, with 30% of uninsured and 46% of insured participants reporting frustration that they are unable to work or contribute, and 25% of uninsured versus 50% of insured participants reporting concern about their job or income.

In multivariable analyses (Table 3), uninsured participants remained about half as likely than insured participants to report all measures of financial distress. Financial distress was often more common at 2–5 years postmetastatic diagnosis than in the first years postmetastatic diagnosis. Lower household incomes were generally associated with greater financial distress, with participants making $15,000–$29,999 annually generally more likely to report distress for example feeling financially stressed (aRR 3.25 [2.36, 4.48]) and being concerned about their job or income (aRR 2.86 [2.10, 3.91]) compared to participants making >$50,000. Age, race, and education were inconsistent predictors of financial distress, with the exception of other race/ethnicity, which was more often negatively associated with financial distress.

**TABLE 3:** Adjusted relative risk of financial distress.

	Out‐of‐pocket medical expenses more than expected		Worry about financial problems in the future		Distressed by not knowing cancer care costs		No choice about the amount of money spent on care		Financially stressed		Worry about financial stress on family		Frustrated that cannot work/contribute		Concerned about job or income	
	RR	95% CI	RR	95% CI	RR	95% CI	RR	95% CI	RR	95% CI	RR	95% CI	RR	95% CI	RR	95% CI
**Uninsured (ref: Insured)**	**0.40**	**[0.31, 0.51]**	**0.55**	**[0.48, 0.64]**	**0.59**	**[0.48, 0.73]**	**0.66**	**[0.52, 0.83]**	**0.46**	**[0.36, 0.59]**	**0.51**	**[0.42, 0.64]**	**0.76**	**[0.62, 0.93]**	**0.53**	**[0.41, 0.67]**
Age (years, ref: 40+)																
Under 40	1.09	[0.93, 1.28]	**0.89**	**[0.80, 0.99]**	1.05	[0.87, 1.26]	1.18	[0.95, 1.46]	1.00	[0.82, 1.21]	0.99	[0.82, 1.21]	0.83	[0.68, 1.02]	0.92	[0.75, 1.13]
Race (ref: White)																
Black	0.96	[0.70, 1.31]	**0.68**	**[0.53, 0.88]**	0.91	[0.66, 1.26]	0.86	[0.61, 1.21]	0.70	[0.46, 1.06]	0.92	[0.65, 1.30]	0.77	[0.53, 1.12]	0.84	[0.57, 1.22]
Hispanic/Latina	**0.36**	**[0.21, 0.61]**	0.83	[0.67, 1.02]	1.05	[0.81, 1.37]	1.14	[0.85,1.52]	**1.36**	**[1.06, 1.76]**	**1.49**	**[1.16, 1.92]**	1.14	[0.85, 1.52]	1.24	[0.94, 1.65]
Other	1.03	[0.87, 1.21]	1.11	[0.99, 1.24]	**0.26**	**[0.16, 0.41]**	**0.23**	**[0.14, 0.37]**	**0.37**	**[0.24, 0.56]**	**0.23**	**[0.14, 0.37]**	**0.34**	**[0.22, 0.51]**	**0.37**	**[0.25, 0.57]**
Years w/metastatic disease (ref:<1)																
1–2 years	**1.71**	**[1.43, 2.03]**	**1.50**	**[1.32, 1.70]**	**1.41**	**[1.21, 1.64]**	0.95	[0.79, 1.16]	**1.53**	**[1.30, 1.81]**	**2.14**	**[1.77, 2.58]**	**2.13**	**[1.72, 2.63]**	**1.40**	**[1.18, 1.66]**
2–5 years	**1.26**	**[1.00, 1.58]**	**1.29**	**[1.11, 1.51]**	0.84	[0.66, 1.09]	1.11	[0.88, 1.40]	1.25	[0.98, 1.59]	**1.67**	**[1.31, 2.13]**	**1.87**	**[1.44, 2.43]**	1.17	[0.91, 1.52]
5+ years	1.37	[0.92, 2.02]	1.11	[0.84, 1.46]	0.90	[0.57, 1.45]	1.06	[0.72, 1.55]	1.12	[0.67, 1.88]	1.05	[0.62, 1.79]	1.29	[0.80, 2.10]	0.87	[0.48, 1.56]
Dependent in Household	0.98	[0.76, 1.28]	0.98	[0.81, 1.17]	**0.55**	**[0.43, 0.69]**	**0.43**	**[0.34, 0.56]**	**0.67**	**[0.51, 0.88]**	**0.59**	**[0.47, 0.73]**	**0.64**	**[0.51, 0.81]**	0.97	[0.71, 1.34]
Annual household income (ref: 50,000+)																
<15,000	0.91	[0.58, 1.42]	0.76	[0.54, 1.08]	1.34	[0.89, 2.02]	0.93	[0.58, 1.48]	**1.66**	**[1.01, 2.73]**	1.14	[0.73, 1.81]	**1.73**	**[1.18, 2.52]**	1.31	[0.71, 2.39]
15,000‐29,999	1.14	[0.90, 1.45]	**1.21**	**[1.03, 1.42]**	**1.82**	**[1.38, 2.39]**	**1.71**	**[1.31, 2.23]**	**3.25**	**[2.36, 4.48]**	**1.39**	**[1.03, 1.88]**	**1.59**	**[1.17, 2.17]**	**2.86**	**[2.10, 3.91]**
30,000‐49,999	**0.74**	**[0.62, 0.89]**	1.09	[0.98, 1.23]	**1.50**	**[1.17, 1.93]**	**1.44**	**[1.14, 1.82]**	**1.75**	**[1.33, 2.31]**	**1.61**	**[1.29, 2.02]**	**1.67**	**[1.32, 2.13]**	**1.71**	**[1.29, 2.26]**
Education (ref: 4‐year college degree)																
High school or less	**1.58**	**[1.24, 2.01]**	0.87	[0.74, 1.02]	**1.43**	**[1.08, 1.91]**	0.40	[0.28, 0.56]	1.06	[0.81, 1.40]	0.98	[0.77, 1.24]	0.92	[0.73, 1.17]	1.03	[0.76, 1.38]
Some college, no degree awarded	1.19	[0.98, 1.44]	1.13	[1.00, 1.27]	**1.56**	**[1.20, 2.04]**	1.24	[1.00, 1.54]	0.86	[0.67, 1.12]	0.89	[0.71, 1.11]	0.79	[0.63, 0.99]	0.78	[0.59, 1.03]
Two‐year degree/vocational school	**1.26**	**[1.04, 1.52]**	1.14	[1.00, 1.30]	**1.48**	**[1.13, 1.96]**	1.14	[0.90, 1.43]	1.08	[0.83, 1.40]	0.93	[0.74, 1.17]	0.55	[0.40, 0.74]	1.00	[0.77, 1.31]
Marital status (ref: unmarried)																
Married, or living with a partner	0.88	[0.73, 1.07]	**0.88**	**[0.78, 0.99]**	**1.32**	**[1.06, 1.65]**	**1.59**	**[1.25, 2.02]**	**1.98**	**[1.51, 2.59]**	**1.46**	**[1.15, 1.85]**	**1.29**	**[1.02, 1.64]**	**1.39**	**[1.06, 1.81]**

*Note*: Adjusted risk ratio from generalized linear model (*N* = 1054). Bolded values represent the relative risk of reporting a domain is significantly different from the referent group at *p* < 0.05.

## 4 DISCUSSION

Our results from a large national survey of young women with metastatic breast cancer point to an alarming degree of cancer‐related financial hardship and significant worry about the financial consequences of their illness. The extent and duration of financial hardship, both in terms of material insecurity and financial psychological distress, reported here is significantly greater than previously reported among insured women with earlier stage disease^18^ and is differentially distributed according to health insurance status. Specifically, uninsured women in our sample reported much greater financial insecurity in terms of ability to pay medical and nonmedical bills, while insured women in our sample reported much greater distress about the cost of their cancer care.

While these findings at first may appear paradoxical, the reality may be that uninsured women with higher material burden are less distressed about their inability to pay because they have grown accustomed to having to shoulder extreme financial burden. By contrast, insured women experiencing unexpected financial shocks may be realizing for the first time that they are, in fact, underinsured. It is also possible that insured women have more asset reserves and wealth to contribute to paying for cancer care but have less resiliency when confronted with unexpected financial loses.

Our results collectively suggest that, when measuring cancer‐related financial hardship, it is imperative to take into account the differences between financial insecurity and financial distress; specifically, focusing only on identifying patients vulnerable to financial toxicity through measurement of high distress or combining measures of insecurity and distress may not accurately capture those who suffer the highest material burden. Conversely, evaluating only material or out‐of‐pocket cost burden may miss patients with high levels of distress who could benefit from psychosocial and financial support to help navigate unfamiliar cost terrain.

Health insurance expansion is a necessary, but insufficient, strategy to address cancer‐related financial burden. Eleven states, to date, have failed to expand Medicaid,^19^ which could substantially improve health care access for a large majority of low‐income, long‐term uninsured women^20^ who are already more vulnerable to being diagnosed with advanced cancer and suffering from higher morbidity and mortality due to lack of access to appropriate screening and early detection services. Studies have suggested that Medicaid expansion has not only significantly improved access to cancer screening and early detection^21^, ^22^, ^23^, ^24^ but also that Medicaid coverage effectively shields individual patients and their families from the large out‐of‐pocket cost burden associated with screening and treatment that uninsured patients would otherwise be expected to “self‐pay”^25^. Even when programs exist to assist “self‐pay” patients, these programs often defray a only small proportion of costs, can be difficult to navigate with strict eligibility criteria and complex application procedures, and require activated and informed patients to follow through.^26^ Medicaid expansion could help reduce the burden of effort required to find and obtain such resources by directly addressing health care bills through insurance provision for those with very low incomes.

More recently, private insurance expansion through Marketplace plans under the Affordable Care Act has also translated into increased access to care and better health outcomes for low‐ and middle‐income Americans.^27^ However, many Americans have been left underinsured and financially exposed by the complexity of private insurance plan design and differences in out‐of‐pocket insurance premiums, coinsurance/copays, and deductibles corresponding to variable coverage generosity. Among insured women with breast cancer, in particular, prior studies have demonstrated that the expansion of high‐deductible health plans and the increasing transference of costs to patients have made even insured patients vulnerable to financial hardship.^6^, ^7^ Therefore, novel value‐based health insurance policy efforts^28^ are needed to ensure minimally sufficient coverage of cancer‐related expenses, including oral medication parity and coverage of medical expenses in the course of clinical trials,^29^, ^30^, ^31^ and interventions are needed to help patients optimize their private insurance coverage, including the engagement of advocates who can assist with denied claims and appeals.^32^ Additional interventions beyond insurance expansion and more generous plan designs are urgently needed to prevent and mitigate cancer‐related financial harm, including expanding health‐related employment protections, offering financial navigation to aid patients in managing their health care bills, identifying resources that can help reduce financial toxicity, and assisting with and streamlining applications.^33^, ^34^ At least five intervention trials funded by the National Cancer Institute are currently underway to address these issues. In the meantime, coping strategies employed by patients with metastatic breast cancer continue to put them at risk and create strain, including stopping treatment, skipping other medical care, not paying nonmedical bills, and making changes to employment—all of which are more common in patients of color.^35^

A number of limitations are noted in this study. First, we acknowledge that our convenience sample drawn from women engaged with the national MBCN is not population‐based and may not be generalizable to all women with advanced breast cancer. In particular, our sample is younger than the average breast cancer patient, and consequently, our results are not likely applicable to the older patients, such as the Medicare population. Additionally, women answering an online survey about breast cancer may be more information seeking than the average patient with advanced cancer; that said, we note that our participants came from diverse geographic areas, income and educational backgrounds, and racial/ethnic groups. It is also likely that women who respond to a survey about financial concerns may have higher financial burden than those who did not answer because the topic is perceived as more salient to them. In addition, all of our measures are self‐reported and may be prone to recall bias or error; for example, the high reported prevalence of debt collections and bankruptcy filings may be an overestimate, reflecting burden perceived as invasive and catastrophic but not necessarily reflecting formal collections and bankruptcy processes. Finally, the cross‐sectional, retrospective nature of this study precluded examination of changes in insurance status and the contributions of specific types of costs over time to financial hardship (e.g., treatment‐specific, indirect vs. direct medical costs). Future studies should examine these issues in cancer survivors longitudinally to better understand how each of these affect financial hardship, which in turn, can better inform interventions to address such stressors.

## 5 CONCLUSION

Our national study provides novel insights about the financial experiences of young women with advanced breast cancer and demonstrates a remarkably high burden of financial insecurity and distress that is differentially distributed according to insurance status. Importantly, uninsured participants with the greatest financial insecurity and the least ability to meet the material demands of their cancer care reported overall less distress and worry about the costs of their care, whereas insured patients who may have met the limits of their insurance coverage in the wake of metastatic disease reported significant distress. These results illustrate the importance of measuring and intervening upon multiple domains of financial toxicity and considering the different nature of hardship that insured versus uninsured patients may face. Interventions such as financial navigation that can help assist with insurance enrollment, maximize insurance utility, and direct patients to additional resources are critically needed, but are not standardized in practice. Finally, macrolevel policy change, including Medicaid expansion, patient‐centered insurance plan design, cost transparency, and cost containment, is urgently needed to prevent and mitigate the financial devastation associated with cancer.

## AUTHOR CONTRIBUTIONS

**Stephanie Wheeler:** Conceptualization (lead); data curation (equal); funding acquisition (equal); investigation (equal); methodology (equal); writing – original draft (lead); writing – review and editing (equal). **Jennifer C. Spencer:** Conceptualization (equal); data curation (equal); formal analysis (equal); methodology (equal); writing – review and editing (equal). **Michelle Manning:** Conceptualization (equal); project administration (lead); writing – review and editing (equal). **Cleo A Samuel:** Conceptualization (equal); writing – review and editing (equal). **Katherine E Reeder‐Hayes:** Conceptualization (equal); writing – review and editing (equal). **Rachel A Greenup:** Conceptualization (equal); writing – review and editing (equal). **Lisa P. Spees:** Conceptualization (equal); writing – review and editing (equal). **Donald Rosenstein:** Conceptualization (equal); funding acquisition (equal); supervision (equal); writing – review and editing (equal).

## FUNDING INFORMATION

We are grateful to the Metastatic Breast Cancer Network for their partnership in identifying study participants, distributing the survey, and for their commitment to reducing the burden of metastatic breast cancer. Funding for this project was provided through the National Comprehensive Cancer Network and Pfizer Independent Grants for Learning & Change (1‐R01‐CA240092‐01, PI: Wheeler and Rosenstein) and by the University of North Carolina's Cancer Care Quality Training Program (T32 CA116339, PI: Basch and Wheeler).

## CONFLICT OF INTEREST STATEMENT

SBW, KRH, and DLR receive grant funding paid to their institution from the Pfizer Foundation. All other authors have no conflicts to report.

## PRECIS

In this national survey, patients with metastatic breast cancer reported an unprecedented level of financial harm, with greater material burden among the uninsured and greater distress about finances among the insured. These results illustrate the shortcomings of health insurance alone as a protective factor against financial distress and highlight the importance of capturing the multidimensional nature of financial toxicity to identify and address financial concerns in diverse populations.

## Supporting information


Table S1.

## Data Availability

Investigators interested in using these samples or accessing the final dataset for future research may do so under the following conditions: (1) IRB approval has been obtained from the institution covering the investigator, (2) data security procedures ensuring patient privacy have been demonstrated by the investigator, and (3) a data use agreement is completed by UNC and the outside investigator. Final datasets for analysis will not include any identifying information.
