# Supplementary material for: Multidimensional financial hardship among uninsured and insured young adult patients with metastatic breast cancer
Source: Cancer Med. 2023 May 6;12(10):11930–40. doi: 10.1002/cam4.5885 (PMC10242847; doi:10.1002/cam4.5885)
Supplement: Supplementary file 1 — Table S1. [file CAM4-12-11930-s001.docx]

**Supplemental Table 1: Financial Toxicity Questions- Financial Distress and Insecurity**

| *Please select a response below indicating your response as it applies to the past 7 days.* | | | | | |
| --- | --- | --- | --- | --- | --- |
| **I know that I have enough money in savings, retirement or assets to cover the cost of my treatment** | | | | | |
|  | Not at All | A little bit | Somewhat | Quite a bit | Very much |
| **My out-of-pocket medical expenses are more than I thought they would be.** | | | | | |
|  | Not at All | A little bit | Somewhat | Quite a bit | Very much |
| **I worry about the financial problems I will have in the future as a result of my illness or treatment** | | | | | |
|  | Not at All | A little bit | Somewhat | Quite a bit | Very much |
| **I feel I have no choice about the amount of money I spend on care** | | | | | |
|  | Not at All | A little bit | Somewhat | Quite a bit | Very much |
| **I am frustrated that I cannot work or contribute as much as I usually do** | | | | | |
|  | Not at All | A little bit | Somewhat | Quite a bit | Very much |
| **I am satisfied with my current financial situation** | | | |  |  |
|  | Not at All | A little bit | Somewhat | Quite a bit | Very much |
| **I am able to meet my monthly expenses** | | | |  |  |
|  | Not at All | A little bit | Somewhat | Quite a bit | Very much |
| **I feel financially stressed** | | |  |  |  |
|  | Not at All | A little bit | Somewhat | Quite a bit | Very much |
| **I am concerned about keeping my job and income, including work at home** | | | | | |
|  | Not at All | A little bit | Somewhat | Quite a bit | Very much |
| **My cancer or treatment has reduced my satisfaction with my present financial situation** | | | | | |
|  | Not at All | A little bit | Somewhat | Quite a bit | Very much |
| **I feel in control of my financial situation** | | | |  |  |
|  | Not at All | A little bit | Somewhat | Quite a bit | Very much |
| **I have been distressed by not knowing what my cancer care cost would be.** | | | | | |
|  | Not at All | A little bit | Somewhat | Quite a bit | Very much |
| **I am worried about the financial stress on my family as a result of my cancer.** | | | | | |
|  | Not at All | A little bit | Somewhat | Quite a bit | Very much |
|  |  |  |  |  |  |
| **Have you ever been contacted by a collection agency as a result of your cancer care?** | | | | | |
|  | Yes | No |  |  |  |
| **Have you ever filed for bankruptcy to manage the cost of your cancer care?** | | | | | |
|  | Yes | No |  |  |  |
